# Passive Tobacco Smoke Exposure, Oxidative Stress and Pediatric Allergic and Obstructive Respiratory Diseases: A Systematic Review and a “Second Oxidative Hit” Hypothesis

**DOI:** 10.3390/antiox15081024

**Published:** 2026-08-17

**Authors:** Bianca Laura Cinicola, Alessandra Gori, Fabrizio Leone, Elia Pignataro, Simone Aloisio, Alessandra Salvatori, Laura Tudini, Caterina Anania, Alberto Spalice, Anna Maria Zicari

**Affiliations:** 1 Department of Maternal Infantile and Urological Sciences, Sapienza University of Rome, 00185 Rome, Italy; alessandra.gori85@gmail.com (A.G.); fabrizio.leone@uniroma1.it (F.L.); elia.pignataro@uniroma1.it (E.P.); laura.tudini@uniroma1.it (L.T.); caterina.anania@uniroma1.it (C.A.); alberto.spalice@uniroma1.it (A.S.); annamaria.zicari@uniroma1.it (A.M.Z.); 2 Department of Radiological, Oncological and Anatomical Pathology Sciences, Sapienza University of Rome, 00185 Rome, Italy; 3 Department of Translational and Precision Medicine, Sapienza University of Rome, 00185 Rome, Italy; 4 Department of Experimental Medicine, La Sapienza University, 00198 Rome, Italy; 5 Department of Human Neurosciences, Sapienza University of Rome, 00185 Rome, Italy; simone.aloisio@uniroma1.it; 6 Pediatrics Clinical Unit, Bambino Gesù Children’s Hospital, IRCCS, 00165 Rome, Italy; alessandra.salvatori94@gmail.com

**Keywords:** passive smoke, secondhand smoke, tobacco, oxidative stress, asthma, allergic rhinitis, NOX2, HDAC2, antioxidant defenses, children

## Abstract

Pediatric allergic and obstructive respiratory diseases are a leading cause of chronic childhood morbidity. Passive tobacco smoke exposure (TSE) is one of the most prevalent and preventable indoor pollutants affecting children, while oxidative stress is increasingly recognized as a key mechanism linking tobacco smoke exposure to airway inflammation. This systematic review critically evaluated the evidence connecting passive TSE to oxidative stress pathways in pediatric airway disease and integrated it into a “second oxidative hit” hypothesis. Following PRISMA 2020 guidelines, PubMed and MEDLINE were searched to identify studies assessing passive TSE, oxidative or antioxidant biomarkers, and respiratory outcomes in children. Of the 77 records identified, four studies met the inclusion criteria. Across the available evidence, passive TSE was associated with increased lipid peroxidation, NOX2 activation, oxidative–inflammatory signaling, depletion of antioxidant defenses, oxidative DNA damage, and impairment of redox-sensitive corticosteroid-response pathways, although findings were not uniform across studies. Clinically, passive TSE children showed persistent allergic rhinitis, greater wheezing severity, poorer asthma control and reduced corticosteroid responsiveness. Despite limited and heterogeneous evidence, the findings provide a biological rationale for a testable “second oxidative hit” hypothesis, whereby passive TSE, combined with a pre-existing inflammatory environment in the airways associated with the underlying disease, could produce an additional oxidative burden. Further prospective studies integrating standardized oxidative biomarkers and objective exposure assessment are needed to validate this hypothesis, and establish temporal and causal relationships, potentially supporting more targeted preventive and personalized strategies.

## 1. Introduction

Pediatric allergic and obstructive respiratory diseases, including asthma, allergic rhinitis, recurrent wheezing disorders and sleep-disordered breathing, represent a major cause of chronic morbidity worldwide and are increasingly associated with long-term respiratory and systemic complications [1,2,3,4,5]. Among these conditions, asthma is the most prevalent chronic respiratory disease in children and is characterized by variable airflow obstruction, airway hyperresponsiveness, mucus hypersecretion and chronic airway inflammation [1,2,4,6]. Persistent airway inflammation and remodeling during childhood may impair lung development and predispose to chronic respiratory disease later in life [2].

In parallel with asthma, allergic rhinitis affects up to 40% of children worldwide and is increasingly recognized as a systemic inflammatory disease rather than a localized upper-airway disorder [3], with reported associations with endothelial dysfunction and increased cardiovascular risk in later life [3]. Similarly, sleep-disordered breathing and obstructive sleep apnea syndrome in children are associated with intermittent hypoxia, autonomic dysfunction, vascular abnormalities and systemic inflammatory activation [5]. Collectively, these observations support the concept that pediatric respiratory diseases extend beyond the airways and may contribute to long-term systemic consequences.

Environmental exposures play a pivotal role in the development and exacerbation of pediatric respiratory diseases. Among them, tobacco smoke remains one of the most important and preventable indoor pollutants affecting children [1,7,8,9]. Epidemiological evidence demonstrates a strong dose-dependent association between passive tobacco smoke exposure (TSE) and asthma onset, wheezing, allergic sensitization and impaired lung function, particularly when exposure occurs during prenatal life or early childhood [10,11,12]. Maternal smoking during pregnancy may interfere with fetal lung maturation through nicotine-mediated oxidative and inflammatory mechanisms, increasing susceptibility to chronic respiratory disorders [2].

Traditional combustible cigarettes contain thousands of toxic compounds—including aldehydes, hydrocarbons, particulate matter, volatile organic compounds, heavy metals and free radicals—capable of inducing airway injury and systemic inflammation [13]. In recent years, increasing attention has focused on alternative products such as heat-not-burn cigarettes (HNBCs) and electronic cigarettes, whose use is rising rapidly among adolescents [14]. Although marketed as reduced-risk alternatives, both generate aerosols containing nicotine, ultrafine particles and aldehydes capable of promoting epithelial injury, oxidative stress and inflammatory responses [15,16]; passive exposure to these emissions may induce oxidative damage in children comparable to that of traditional cigarette smoke [8,13].

Oxidative stress is increasingly recognized as a key mechanism linking passive TSE to airway inflammation and systemic injury [1,2,4,7]. It results from an imbalance between reactive oxygen species (ROS) and antioxidant defense systems, leading to damage of lipids, proteins, DNA and cellular structures [7]. In pediatric asthma and allergic diseases, activated eosinophils, neutrophils, macrophages and airway epithelial cells are major endogenous sources of ROS [1,4,7], while environmental pollutants, allergens and cigarette smoke directly stimulate ROS generation or amplify inflammatory pathways that further increase oxidative burden [1,2,4,7]. Excessive ROS contribute to epithelial-barrier dysfunction, mucus hypersecretion, airway hyperresponsiveness and remodeling [1,2,4,7], and activates redox-sensitive transcription factors (NF-κB, AP-1) that promote the release of pro-inflammatory cytokines and perpetuate chronic airway inflammation [4,7]. Cigarette smoke also impairs endogenous antioxidant pathways, including Nrf2-mediated responses, amplifying oxidative injury and contributing to corticosteroid resistance in severe asthma [1,7]. NADPH oxidase activation, particularly the NOX2 isoform, appears to be a major molecular pathway linking TSE to ROS overproduction and systemic oxidative damage [3,8,17].

Importantly, the detrimental effects of passive TSE are not confined to the respiratory tract. Chronic airway inflammation and oxidative stress may evolve into a systemic inflammatory condition associated with endothelial dysfunction, platelet activation, vascular injury and increased cardiovascular risk later in life [3,8,13,17].

Despite growing interest, the molecular mechanisms connecting airway inflammation to systemic oxidative injury in passive TSE children remain incompletely understood and poorly defined. The aim of this systematic review is therefore to critically summarize current evidence on the role of passive TSE in pediatric allergic and obstructive respiratory diseases, focusing on the interplay between oxidative stress and airway inflammation, and to integrate the available evidence into a unifying “second oxidative hit” model.

## 2. Materials and Methods

This systematic review was conducted and reported according to the Preferred Reporting Items for Systematic Reviews and Meta-Analyses (PRISMA 2020) guidelines. The review aimed to evaluate the association between passive TSE and oxidative stress pathways in pediatric allergic and obstructive respiratory diseases, with particular focus on oxidative stress biomarkers, inflammatory mechanisms and clinical respiratory outcomes. Because of the substantial heterogeneity in study design, biomarker selection, biological matrices and outcome reporting, a formal quantitative meta-analysis was not considered methodologically appropriate. Observational, cohort, cross-sectional and case–control studies, and translational/mechanistic investigations were included. Among cross-sectional studies, case–controls were defined as patients with respiratory disease versus healthy subjects, while studies were defined as comparative when intra-group patients (TSE versus non-TSE) were considered.

Review articles were excluded from the primary qualitative synthesis; however, they were examined for additional references and contextual interpretation. Studies were eligible if they investigated children or adolescents with allergic or obstructive respiratory conditions, assessed passive exposure to tobacco smoke (second- and thirdhand smoke), encompassed both traditional and heated tobacco products, and analyzed oxidative stress factors.

Exclusion criteria: Adult-only populations; animal or in vitro studies; non-English language studies; studies without oxidative stress-related outcomes; studies lacking pediatric respiratory disease populations; and conference abstracts without sufficient methodological information.

The PICO statement was as follows:

Population (P): Children or adolescents with asthma, recurrent wheezing, allergic rhinitis, obstructive sleep apnea syndrome (OSAS), sleep-disordered breathing (SDB) or related allergic/non-allergic obstructive airway diseases.

Intervention or Exposure (I): Studies evaluating passive TSE, environmental tobacco smoke, secondhand or thirdhand smoke, parental smoking, cotinine/nicotine exposure, or passive exposure to heated tobacco products or electronic cigarettes.

Comparison or control (C): Non-exposed children’s cohorts or inter group patients.

Outcome (O): Studies reporting at least one oxidative stress biomarker, antioxidant defense marker, and oxidative–inflammatory pathway, associated with allergic/ non allergic obstructive respiratory diseases.

### 2.1. Search Strategy

A comprehensive literature review was conducted utilizing PubMed and MEDLINE databases. The search strategy was implemented in March 2026, employing controlled vocabulary and pertinent keywords related to passive TSE, oxidative stress, pediatric populations, and allergic or obstructive respiratory diseases, within TITLE/ABSTRACT (tiab) fields.

A comprehensive literature search was performed using the following search strategies:

Strategy 1: (“secondhand smoke”[tiab] OR “second-hand smoke”[tiab] OR “environmental tobacco smoke”[tiab] OR “passive smoking”[tiab] OR “passive smoke exposure”[tiab] OR “tobacco smoke exposure”[tiab] OR “tobacco smoke pollution”[tiab] OR cotinine[tiab] OR nicotine[tiab] OR smoking[tiab] OR smoke[tiab] OR “thirdhand smoke”[tiab] OR “third-hand smoke”[tiab]) AND (“oxidative stress”[tiab] OR oxidant*[tiab] OR antioxidant*[tiab] OR “redox”[tiab] OR “reactive oxygen species”[tiab] OR ROS[tiab] OR “8-isoprostane”[tiab] OR isoprostane*[tiab] OR “malondialdehyde”[tiab] OR MDA[tiab] OR glutathione[tiab] OR “superoxide dismutase”[tiab] OR SOD[tiab] OR catalase[tiab] OR “total antioxidant capacity”[tiab] OR “nitric oxide”[tiab] OR nitrosative[tiab]) AND (child*[tiab] OR adolescen*[tiab] OR pediatric*[tiab] OR paediatric*[tiab] OR infant*[tiab] OR preschool*[tiab]) AND(asthma[tiab] OR wheez*[tiab] OR “allergic rhinitis”[tiab] OR rhinitis[tiab] OR “sleep apnea”[tiab] OR “sleep apnoea”[tiab] OR “obstructive sleep apnea”[tiab] OR “obstructive sleep apnoea”[tiab] OR OSAS[tiab] OR OSA[tiab] OR “sleep disordered breathing”[tiab] OR SDB[tiab]).

Filters applied: Adaptive Clinical Trial, Clinical Study, Clinical Trial, Clinical Trial, Phase I, Clinical Trial, Phase II, Clinical Trial, Phase III, Clinical Trial, Phase IV, Collected Work, Comparative Study, Controlled Clinical Trial, Dataset, Electronic Supplementary Materials, Equivalence Trial, Evaluation Study, Introductory Journal Article, Letter, Meta-Analysis, Multicenter Study, Network Meta-Analysis, Observational Study, Pragmatic Clinical Trial, Randomized Controlled Trial, Systematic Review, Validation Study, English, Humans.

Strategy 2: (“Tobacco Smoke Pollution”[Tiab] OR “cotinine” [Tiab] OR “nicotine” [Tiab] OR “tobacco” [Tiab] OR “smoke” [Tiab]) AND (“oxidative stress” [Tiab]) AND (“Child” [Tiab] OR “Adolescent”[Tiab] OR “pediatric*”[Tiab] OR “child” [Tiab] OR “adolescent*”[Tiab] OR “children” [Tiab]) AND (“Asthma”[Tiab] OR “wheez*”[Tiab] OR “Rhinitis, Allergic”[Tiab] OR “allergic rhinitis” [Tiab] OR “Sleep Apnea Syndrome*”[Tiab] OR “obstructive sleep apnea” [Tiab] OR “OSAS” [Tiab] OR “sleep disordered breathing” [Tiab] OR “SDB” [Tiab])”.

Reference lists of eligible studies and relevant reviews were screened manually to identify additional studies.

### 2.2. Study Selection and Data Extraction

Two reviewers (B.L.C. and A.G.) independently screened titles and abstracts; full texts of potentially relevant studies were then assessed, with disagreements resolved by consensus. Data extraction was performed independently by two reviewers using a standardized framework including study and population characteristics, smoke-exposure assessment, oxidative stress biomarkers, and clinical outcomes.

### 2.3. Risk-of-Bias Assessment

Because the included studies are heterogeneous and mostly observational, the risk of bias was evaluated according to study design. Cross-sectional studies were assessed using the Joanna Briggs Institute (JBI) Critical Appraisal Checklist, while cohort and case–control studies were evaluated with the Newcastle–Ottawa Scale (NOS). Studies were graded as having low, moderate or high risk of bias.

## 3. Results

### 3.1. Study Selection and Characteristics

**Figure 1:**
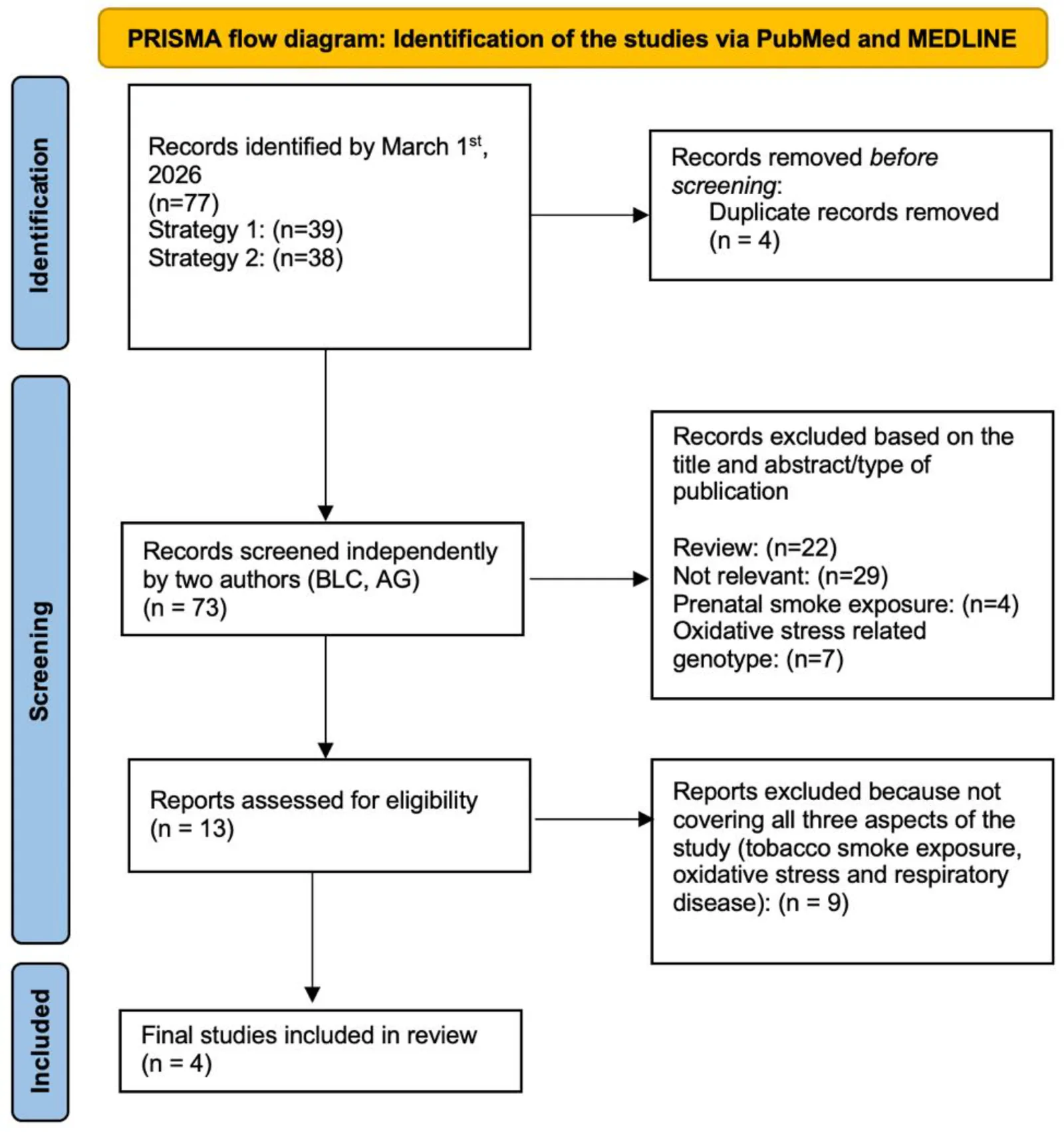
PRISMA flow diagram. Modified from [18].

The systematic literature search identified 77 records through database searching. After removal of duplicates and screening, four studies were considered relevant for qualitative synthesis (Figure 1).

The included studies were published between 2014 and 2018 and conducted across heterogeneous geographic settings in Europe, North America and Asia. Most adopted observational designs—predominantly cross-sectional or cohort-based—although mechanistic translational and biomarker-focused investigations were also represented. Sample sizes ranged from 19 children in the bronchoalveolar lavage (BAL) mechanistic study to 160 participants in the largest included study. The analyzed pediatric populations included children with asthma of varying severity, recurrent wheezing, and allergic rhinitis.

Smoke-exposure assessment varied substantially: two studies relied on parental questionnaires or household smoking history, whereas the other two incorporated objective biomarkers such as serum/plasma or urinary cotinine, and a subset differentiated low- from high-level passive TSE using predefined cotinine thresholds. Oxidative stress was evaluated in heterogeneous biological matrices including serum, urine, BAL fluid, and nasal lavage. The investigated biomarkers included malondialdehyde (MDA), glutathione, superoxide dismutase (SOD), 8-oxo-7,8-dihydro-2′-deoxyguanosine (8-oxo-dG), isoprostanes and NOX2-derived peptides, together with inflammatory mediators associated with oxidative signaling such as IL-8, IL-17, myeloperoxidase (MPO) and matrix metalloproteinases. Despite this heterogeneity, three studies consistently reported increased oxidative burden or impaired antioxidant defenses in TSE children with respiratory disease, while a study detecting specific biomarkers on nasal lavage failed to identify a relevant association between oxidative stress, inflammation and severity of respiratory disease in passive TSE versus non-TSE children. The characteristics, biological role and direction of evidence of the included studies are summarized in Table 1.

### 3.2. Passive Smoke Exposure and Lipid Peroxidation Pathways

Evidence from two studies supported an association between passive TSE and increased lipid peroxidation in pediatric airway disease. Investigations demonstrated elevated biomarkers of oxidative membrane injury in TSE children, including increased MDA, isoprostanes and NOX2-related oxidative activity. In children with persistent allergic rhinitis, passive TSE was associated with enhanced NOX2 activation (soluble NOX2-derived peptide) [3]. Similarly, a study in severe asthmatic children showed that MDA in BAL fluid increased in association with TSE [19].

The recurrence of lipid-oxidation biomarkers across heterogeneous biological matrices supports a coherent oxidative response induced by environmental smoke exposure in susceptible children. These results showed a predominantly positive association between passive TSE and lipid-peroxidation markers.

### 3.3. Oxidative DNA Damage and Systemic Redox Burden

Oxidative nucleic-acid damage emerged as a further pathway. A study evaluating urinary 8-oxo-dG demonstrated associations between TSE and oxidative DNA injury in children with asthma, with increased DNA-damage markers linked to the specific clinical phenotype of intermittent asthma [21]. These observations support the hypothesis that smoke-related oxidative stress in pediatric airway disease extends beyond transient inflammatory responses to contribute to a broader systemic oxidative burden affecting nucleic-acid integrity.

### 3.4. Oxidative Inflammation and Airway Remodeling

Passive TSE was associated with increased neutrophilic inflammatory signaling and included alterations in IL-8, IL-17, MPO, MMP-9/TIMP-1, CXCL8 and NOX2-related oxidative activation. In recurrent-wheezing children, passive TSE was associated with more severe respiratory symptoms and evidence of airway inflammatory activation, although biomarkers did not show statistically significant between-group differences [20]. A mechanistic study in severe asthma demonstrated enhanced oxidative–inflammatory signaling within BAL macrophages of TSE children, supporting the concept of persistent airway inflammatory activation induced by passive smoking [19].

### 3.5. Corticosteroid Resistance and Redox-Sensitive Pathways

One of the most mechanistically relevant findings concerned oxidative pathways linked to corticosteroid resistance. In children with severe asthma exposed to passive smoke, reduced histone deacetylase-2 (HDAC2) activity and expression were observed together with activation of PI3K/Akt signaling and increased oxidative stress markers [19]. These molecular abnormalities were associated with impaired corticosteroid responsiveness and poorer asthma control, providing biologically plausible evidence that passive TSE can directly interfere with redox-sensitive anti-inflammatory regulatory pathways and may thereby contribute to severe, treatment-refractory asthma phenotypes.

### 3.6. Antioxidant-System Impairment

Another finding involved impairment of enzymatic and non-enzymatic antioxidant defense systems. Children with asthma demonstrated significantly lower levels of the antioxidant molecule superoxide dismutase in serum, indicating that passive TSE may contribute not only to oxidant generation but also to depletion or dysregulation of antioxidant defenses, thereby amplifying airway susceptibility to inflammatory injury [21]. A study investigating recurrent wheezing additionally evaluated nasal glutathione in relation to passive TSE; wheezing children exposed to passive smoke generally showed worse respiratory symptom severity although biomarkers did not consistently differ by exposure intensity [20].

### 3.7. Clinical Respiratory Outcomes

Across the included studies, passive TSE was associated with worse respiratory clinical outcomes. TSE children showed persistent allergic rhinitis [3] increased wheezing severity [20], intermittent asthma [21], poorer asthma control and reduced responsiveness to therapy [19]. Taken together, the available evidence supports a clinically meaningful association between passive TSE, oxidative-stress amplification and respiratory disease severity in pediatric allergic and obstructive airway disorders.

### 3.8. Risk of Bias

The main recurrent limitations across studies included small sample sizes, heterogeneity of oxidative biomarkers, inconsistent smoke-exposure ascertainment, variable adjustment for socioeconomic and environmental confounders, and a general lack of longitudinal oxidative-stress measurements. Nevertheless, several studies strengthened internal validity through objective cotinine measurement, repeated biomarker sampling, mechanistic translational analyses, airway-specific biological matrices and integration of clinical severity outcomes. Preliminary risk-of-bias judgments are summarized in Table 2.

A complementary study-level assessment of the main methodological limitations, including their likely direction and magnitude and their potential impact on the conclusions of this review, is provided in Table 3. Direction and magnitude of bias and its impact on review conclusions were qualitatively judged according to study design, exposure and outcome assessment, statistical precision, and potential confounding; these ratings do not represent quantitative bias-adjusted estimates.

## 4. Discussion

This systematic review synthesizes a limited but consistent body of evidence linking passive TSE to oxidative stress and clinical severity in pediatric allergic and obstructive respiratory disease. Despite a low number of scientific articles focused on this topic, marked heterogeneity in design, biomarkers and biological matrices, the included studies demonstrate that passive TSE increases oxidative–inflammatory signaling, depletes antioxidant defenses, and amplifies and interferes with redox-sensitive corticosteroid-response pathways. One of the selected studies failed to find an association between smoking and oxidative stress; however, it is the only study to have analyzed inflammatory and oxidative/antioxidant markers in nasal fluid [20]. These results may have been affected by the potentially low quantity of biomarkers in this biological fluid, making it difficult to establish an association with passive TSE.

Among the diseases considered, asthma with various levels of severity, recurrent wheezing and persistent allergic rhinitis have been analyzed. Although a preliminary study demonstrated increased oxidative stress in children with OSAS [5], no studies have considered smoke exposure as an amplifier of inflammatory response and oxidative stress exacerbation of severity in OSAS.

Analysis of the selected studies identified three mechanisms underlying the proposed approach.

First, continuous exposure to allergens stimulates nasal eosinophils to produce ROS [22]. NADPH oxidase activity represents a source of excessive ROS production and inflammation. Previous studies have shown that NADPH oxidase isoforms NOX1, NOX4, DOX1, and DOX2 are overexpressed in the airway epithelium and mucosa [23,24].

In allergic rhinitis, it was demonstrated that NOX2 activation was exacerbated by passive smoke, with increased soluble NOX2-derived peptide and isoprostanes [3].

Oxidative consequences of passive smoking are not confined to the airway but extend to the endothelium—an early antecedent of later cardiovascular risk. Indeed, Nicotinamide-adenine dinucleotide phosphate (NADPH) oxidase is a primary source of cellular superoxide anion production in humans [25], and studies performed in animals and humans suggest that NADPH oxidase activity modulates arterial tone [26]. Accordingly, it has been shown that a lack of or deficient NADPH oxidase isoform 2 (NOX2) activity is associated with arterial dilation in humans [27]. Children affected by persistent allergic rhinitis and exposed to passive smoking, compared to those who are non-exposed, had significantly higher sNOX2-dp, serum 8-iso-PGF2a and also lower FMD, an early sign of endothelial dysfunction [3].

Second, a broad decrease in enzymatic and non-enzymatic antioxidant defenses is observed in asthma, as previously demonstrated [28], and depletion of the enzyme SOD reflects impaired antioxidant defenses, reducing the capacity to counteract cigarette smoke-induced oxidative stress in subjects with asthma and eczema [21].

A nutritional dimension reinforced this mechanism: although it was not possible to include it among the selected studies because oxidative stress markers were not measured, a large cross-sectional analysis showed that a higher dietary antioxidant quality score attenuated the association between passive TSE and asthma outcomes, suggesting that endogenous and dietary antioxidant capacity can modulate susceptibility [29].

The potential involvement of impaired antioxidant defenses is further supported by pediatric observational evidence beyond the studies included in the systematic synthesis. Preston et al. reported lower plasma ascorbate concentrations in children exposed to environmental tobacco smoke, objectively assessed by urinary cotinine, despite comparable vitamin C intake [30]. Similarly, Wilson et al. found inverse associations between serum cotinine and several antioxidant micronutrients, including vitamin C, carotenoids, folate, and vitamin A [31]. These findings provide a biological rationale for investigating whether restoration of antioxidant status may mitigate TSE-associated oxidative alterations. However, they do not establish a therapeutic benefit of supplementation, which requires dedicated pediatric intervention studies assessing efficacy, dosing, and safety.

Third, HDAC2 impairment via PI3K/Akt activation provides a direct molecular bridge between oxidative stress and corticosteroid resistance, offering a possible explanation for the treatment-refractory phenotypes observed in TSE children with severe asthma [19].

Additional experimental evidence suggests that redox-sensitive inflammatory pathways may complement the mechanisms identified in the included studies. Cigarette smoke-induced oxidative injury has been linked to DAMP/RAGE signaling and to TLR4/MyD88/TRAF6-dependent NADPH oxidase activation, with downstream MAPK and NF-κB signaling and pro-inflammatory mediator production [32,33]. These pathways are consistent with the broader evidence linking tobacco smoke-induced oxidant–antioxidant imbalance to airway inflammation [34] and may provide additional mechanistic support to the direct evidence derived from the systematic synthesis. Their relevance to passive TSE in children, however, remains to be specifically investigated.

The leukotriene pathway provides additional clinical insight. Among the studies that were not selected because they did not strictly address the research question, two papers demonstrated that urinary LTE4 identified TSE asthmatic children at higher risk of severe exacerbations requiring emergency department or urgent-care visits, reflecting the high inflammatory state caused by asthma and exacerbated by smoke. Consistent with this, leukotriene-pathway studies showed that passive TSE modifies the relationship between urinary LTE4, treatment response and exacerbation risk—possibly identifying LTE4 as a redox-sensitive susceptibility biomarker with direct therapeutic implications [35,36,37].

All these findings may be interpreted within a hypothesis-generating framework, the “second oxidative hit” model: repeated passive TSEs, combined with a pre-existing inflammatory environment in the airways associated with the underlying disease, could produce an additional oxidative burden that, in turn, could contribute to less effective disease control and a reduced response to treatment. This hypothesis could be further explored through three complementary research approaches. First, objective exposure to TSE should be assessed repeatedly in prospective longitudinal cohorts, preferably using cotinine-based measurements across different matrices, along with oxidative/antioxidant biomarkers to determine whether cumulative or persistent exposure predicts subsequent changes in the redox balance. Second, serial clinical assessments of disease control, exacerbations, and response to corticosteroids should be conducted in the same cohorts to determine whether oxidative alterations precede and predict worsening respiratory outcomes. Third, the reversibility of oxidative damage should be evaluated through lifestyle interventions aimed at reducing exposure or quitting smoking, to determine whether objectively measured reductions in TSE are accompanied by parallel decreases in oxidative burden and improvements in clinical outcomes. Taken together, these study designs would allow for a more rigorous assessment of temporal relationships, dose–response relationships, and reversibility than is possible with the currently available cross-sectional evidence.

Background and contextual studies broaden the framework. Although oxidative stress biomarkers demonstrate the biological impact of TSE, genetic and epigenetic determinants capable of modulating oxidative stress responses provide a biological basis for interindividual susceptibility to tobacco smoke-related respiratory disease.

In this context, the search strategy used to select the previous studies identified an additional 10 studies.

All included studies specifically evaluated passive TSE as the environmental trigger and investigated how oxidative stress-related genetic, epigenetic or antioxidant pathways modify the risk of respiratory disease development or progression.

Eight studies examined genetic susceptibility through polymorphisms in oxidative stress-related genes, whereas two evaluated epigenetic mechanisms associated with prenatal TSE.

Although the magnitude of the observed associations varied among studies, most reported that oxidative stress-related genetic susceptibility became evident primarily following prenatal or postnatal TSE rather than in its absence. Overall, TSE was consistently evaluated as the primary environmental exposure, while oxidative stress-related genetic, epigenetic, or antioxidant pathways were investigated as potential modifiers of individual susceptibility. The molecular pathways most frequently implicated involved the glutathione S-transferase family (GSTM1, GSTT1, and GSTP1) and the antioxidant response regulator NFE2L2, while recent evidence also highlighted the contribution of epigenetic mechanisms, including altered DNA methylation and microRNA expression. Collectively, these molecular alterations were associated with impaired lung development, wheezing, asthma susceptibility, reduced pulmonary function and, in some studies, increased disease severity [38,39,40,41,42,43,44,45,46,47].

Taken together, the evidence reviewed supports a coherent pathogenic framework in which oxidative stress represents a central biological mechanism linking TSE during both prenatal and postnatal life to the development and progression of pediatric respiratory diseases. Whether preconception parental or ancestral tobacco exposure may further modify offspring susceptibility through germline epigenetic mechanisms remains an emerging research question requiring specifically designed multigenerational studies.

With respect to emerging products, review-level evidence supports the biological plausibility of e-cigarette- and heat-not-burn cigarette-related oxidative injury [15,16], yet direct pediatric data on passive exposure to electronic and heat-not-burn products are scarce [8]. Unlike conventional cigarettes, e-cigarettes do not involve tobacco combustion and have a different toxicant profile; nevertheless, their aerosols may contain reactive oxygen species, carbonyl compounds, ultrafine particles, and other potentially harmful constituents [48]. Experimental evidence further indicates that aerosol components, including propylene glycol, vegetable glycerin, and flavoring chemicals, are not necessarily biologically inert and may impair airway epithelial function and promote oxidative and inflammatory responses [49,50,51,52]. Thus, although conventional tobacco smoke and e-cigarette aerosols differ in composition and toxicant burden, oxidative stress and airway inflammation may represent partially convergent biological responses. However, these mechanisms have been characterized predominantly in experimental models and active users, and their relevance to passive exposure in children remains to be established.

### 4.1. Methodological Considerations

The cross-sectional design of the included studies precludes establishing temporality or causality between passive TSE, oxidative alterations, and respiratory outcomes. Although two studies [3,19] compared TSE and non-TSE children within the same clinically defined respiratory condition, and objective cotinine assessment reduced exposure misclassification in one of the mechanistically informative studies [3], reverse causation cannot be entirely excluded. Accordingly, the proposed “second oxidative hit” model requires prospective longitudinal validation.

A further limitation is the reliance on single-time-point measurements, which cannot capture the dynamic nature of TSE and oxidative/antioxidant balance, and heterogeneity in TSE ascertainment, which varied across parental reports, cotinine measurements, biological matrices, exposure definitions, and thresholds. Questionnaire-based assessment may be affected by recall or social-desirability bias and under-reporting, potentially leading to exposure misclassification, although its direction and magnitude cannot be reliably determined. Conversely, cotinine provides a more objective measure of recent exposure but does not necessarily reflect cumulative TSE. Given the small number of eligible studies and methodological heterogeneity, stratified or sensitivity analyses according to exposure-assessment method were not considered informative.

Residual confounding should also be considered, as socioeconomic factors, co-exposure to other pollutants, dietary antioxidant intake, and other environmental determinants were not consistently accounted for and may have influenced the observed associations. Therefore, TSE should be interpreted as a potential additional oxidative burden within a multifactorial environmental and biological context.

Future longitudinal studies should therefore incorporate repeated within-subject assessments of objective TSE and oxidative/antioxidant balance, while adequately accounting for relevant potential confounders. Assessments should include clinically informative periods such as stable disease, respiratory infections or exacerbations, recovery, and, where feasible, different seasons, while balancing methodological requirements with the feasibility and invasiveness of repeated sampling in pediatric populations.

### 4.2. Limitations and Strengths

The limitations are substantial and largely inherited from the primary evidence: the number of eligible studies is small, they are cross-sectional with modest samples, longitudinal oxidative measurements are absent, and biomarker methods and exposure ascertainment are heterogeneous.

From a clinical perspective, current evidence does not support the routine use of oxidative stress biomarkers such as 8-oxo-dG or sNOX2-dp for pediatric risk assessment, given the limited and heterogeneous data, lack of standardized pediatric reference ranges and prognostic thresholds, and methodological variability across assays. Further validation is therefore required before their clinical implementation. Conversely, objective TSE assessment by urinary or salivary cotinine is readily available and may provide a more practical tool for identifying and monitoring exposure, particularly in children with severe or difficult-to-control respiratory disease.

Another limitation is that, despite the inclusion of several biomarkers specific to exposure and oxidative stress and the use of two search strategies—one more specific to biomarkers and one more general—they may not have captured all individual tobacco smoke constituents or their metabolites potentially relevant to oxidative and respiratory effects. These include acrolein, a highly reactive unsaturated aldehyde abundant in tobacco smoke, which can promote airway epithelial injury, oxidative stress, and inflammatory responses [53], and its major urinary metabolite 3-hydroxypropylmercapturic acid (3-HPMA), an established biomarker of acrolein exposure [54]. Therefore, studies specifically focused on these markers may not have been fully captured.

However, this systematic review has strengths. First, to our knowledge, it is the first systematic review specifically investigating the relationship between passive TSE oxidative stress, and disease severity in pediatric allergic and obstructive respiratory disorders. This study synthesizes clinical, biochemical, and mechanistic evidence, to provide the first comprehensive overview of the biological pathways linking passive TSE to respiratory disease progression in children.

A further strength is that the review clearly identifies a substantial gap in the current literature. Despite the well-established detrimental effects of passive TSE on pediatric respiratory health, only a limited number of studies have specifically investigated oxidative stress as the mechanistic link between passive TSE and disease severity. This finding highlights how underexplored this field remains and emphasizes the need for dedicated research using standardized methodologies and clinically relevant oxidative stress biomarkers.

## 5. Conclusions

The four studies included in the systematic review provide evidence of associations between passive exposure to TSE, alterations in oxidative/antioxidant pathways, and the clinical characteristics and outcomes of certain pediatric airway diseases. These findings have led us to propose the “second oxidative hit” as a unifying hypothesis, according to which repeated passive exposure to TSE may represent an additional exogenous oxidative insult that overlaps with the pre-existing pro-oxidant environment of inflamed pediatric airways and may, in turn, contribute to less effective disease control and a reduced response to treatment. The oxidative and redox-sensitive pathways identified in the included studies, together with the broader mechanistic, genetic, and epigenetic evidence discussed in this review, support the biological plausibility of this hypothesis. However, this interpretation must be considered in light of the limited number of studies, their predominantly cross-sectional design, the small and heterogeneous populations, and the variability in the assessment of TSE and oxidative biomarkers—factors that preclude drawing definitive causal conclusions.

From a translational perspective, preventing and reducing exposure to secondhand tobacco smoke remains the primary feasible strategy for children with respiratory diseases. Future research should prospectively test the “second oxidative hit” hypothesis and its clinical relevance. Longitudinal cohorts combining repeated objective assessments of TSE, preferably using cotinine-based measurements, with oxidative/antioxidant biomarkers could determine whether cumulative or persistent exposure predicts subsequent changes in the redox balance and whether such alterations precede worsening disease control, exacerbations, or a reduction in or diminished response to corticosteroids. Complementary, family-based interventions aimed at reducing exposure to smoke could assess reversibility by determining whether reductions in objectively measured TSE are accompanied by parallel improvements in oxidative biomarkers and clinical outcomes. These approaches would allow for a more rigorous examination of temporality, dose–response relationships, and partial reversibility than is possible with the currently available cross-sectional evidence. Future studies should also address the issues of individual susceptibility, under-studied pediatric respiratory conditions, and passive exposure to emerging nicotine-containing products. Strengthening the evidence in these areas could support the development of more targeted preventive strategies and personalized treatments, while also providing a more solid scientific basis for public health policies aimed at protecting children from secondhand smoke exposure.

## Figures and Tables

**Table 1:** Characteristics and biological contribution of the included studies (*n* = 4).

Study	REF N.	Design	N Cases and Controls	Population (Disease)	Key Biomarkers of Oxidative Stress and Smoke Metabolites/Type of Sample	Results	Outcome
Loffredo 2018	[3]	Cross-sectional case–control study	65 cases (32 TSE vs. 33 non-TSE) vs. 65 healthy controls	Persistent allergic rhinitis	Serum sNOX2-dp, isoprostanes, NO bioavailability; plasma cotinine	Allergic rhinitis group vs. healthy controls: ↑ serum sNOX2-dp and 8-iso-PGF2a; subgroup analysis in the allergic rhinitis group of TSE vs. non-TSE: ↑ sNOX2-dp ↑ serum 8-iso-PGF2a	Passive TSE was associated with increased NOX2-driven oxidative stress in persistent allergic rhinitis
Kobayashi 2014	[19]	Cross-sectional comparative study; mechanistic	19 subjects (10 not TSE and 9 TSE)	Uncontrolled severe asthma	BAL cell count, MDA, HDAC2 protein/activity, PI3K/Akt, CXCL8; saliva or urine cotinine;	Subjects in the TSE group vs. non-TSE group: in BAL ↑ neutrophils, ↓ eosinophils, ↑ MDA, ↑ CXCL8; positive correlation between CXCL8 concentrations and the percentage of neutrophils; in AMs of TSE BAL, ↓ HDAC2 protein level, ↓ activity of IP-HDAC2, ↑ HDAC2 and Akt1 phosphorylation. Akt1 phosphorylation levels were positively correlated with HDAC2 phosphorylation; Dexamethasone at 10–6 M leads to ↓ TNFa-induced CXCL8 production by 40% in AMs	Passive TSE was associated with increased oxidative stress and inflammatory markers and impairs HDAC2 expression and activity via PI3K/Akt, a pattern consistent with steroid resistance in severe asthma
Yilmaz 2018	[20]	Cross-sectional comparative study	160 subjects (125 TSE-91 High-level TSE and 24 Low-level TSE vs. 35 non-TSE)	Recurrent wheezing	Nasal lavage glutathione, IL-8, IL-17, MMP-9 and TIMP-1; serum cotinine and SP-D	Serum cotinine levels between 3 and 12 ng/mL, and above 12 ng/mL were defined as lower and higher level TSE, respectively. Wheezing symptom scores were higher in TSE children. No differences in the level of biomarkers in the TSE and non-TSE groups and between the two groups of TSE children	Passive TSE was associated with worse wheezing severity; no relevant association between biomarkers of oxidative stress inflammation and severity of respiratory disease.
Topic 2017	[21]	Cross-sectional comparative study	96 subjects (48 TSE in both prenatal and postnatal period vs. 48 non-TSE)	Asthma: 10.4% intermittent, 89.6% persistent (40.6% mild persistent, 49% severe persistent)	Urinary 8-oxo-7,8-dihydro-2-deoxyguanosine; serum MPO and Cu/Zn-superoxide dismutase (SOD)	↑ levels of urinary 8-oxo-7,8-dihydro-2-deoxyguanosine in children with TSE intermittent asthma, in comparison to the non-TSE children with intermittent asthma and to children with mild and severe persistent asthma, regardless of TSE. The level of serum SOD was altered in asthmatic children with eczema under the influence of TSE. Increased urinary 8-oxodG and TSE were identified as independent risk factors associated with intermittent asthma	Passive TSE and increased 8-oxo-dG were linked to intermittent asthma clinical manifestations

**Table 2:** Preliminary risk-of-bias appraisal of core studies.

Study	Design	Exposure Assessment	Main Limitation	Preliminary Risk
Loffredo 2018 [3]	Cross-sectional	Cotinine/smoking history	Single-time-point biomarkers.	Moderate
Kobayashi 2014 [19]	Cross-sectional Mechanistic	Cotinine/smoking history	Very small cohort. Single-time-point biomarkers.	Moderate-to-high
Yilmaz 2018 [20]	Cross-sectional	Cotinine	Single-time-point biomarkers.	Moderate
Topic 2017 [21]	Cross-sectional	Questionnaire-based TSE	Self-reported exposure. Single-time-point biomarkers.	Moderate

**Table 3:** Systematic assessment of limitations of included studies.

Study	Main Source(s) of Bias/Limitation	Likely Direction/Magnitude	Impact on Review Conclusions	Rationale
Loffredo 2018 [3]	Residual confounding; single-time-point assessment; single-center population	Uncertain; moderate	Medium	Cross-sectional assessment limits causal inference, although objective cotinine measurement reduces exposure misclassification and comparison within a clinically defined population improves group comparability. Temporality and cumulative exposure cannot be established.
Kobayashi 2014 [19]	Small, highly selected severe-asthma population; limited control of confounding	Uncertain; potentially large	Medium	The small sample, clinical selection and cross-sectional design limit causal and clinical inference. However, exposed and unexposed children had comparable severe-asthma characteristics, and the study provides detailed mechanistic evidence.
Yilmaz 2018 [20]	Limitations of nasal lavage and single-time-point biomarker assessment; exposure categorization	Mainly toward null for biomarker associations; magnitude uncertain	Medium	Sampling limitations may have reduced sensitivity to detect oxidative differences, although exposure was objectively assessed by cotinine and biomarker outcomes were laboratory-determined. A true null association cannot be excluded.
Topic 2017 [21]	Questionnaire-based TSE assessment; residual confounding; limited subgroup precision	Uncertain; moderate	Medium	Exposure misclassification and subgroup imprecision reduce confidence in specific associations, while assessment of multiple oxidative/antioxidant markers provides complementary evidence on redox alterations.

## Data Availability

No new data were created or analyzed in this study. Data sharing is not applicable to this article.

## Abbreviations

The following abbreviations are used in this manuscript:

TSE	tobacco smoke exposure
NOX2	NADPH oxidase 2
PGF2a	prostaglandin F2α
HDAC2	histone deacetylase-2
BAL	bronchoalveolar lavage
AM	alveolar macrophages
TIMP-1	tissue inhibitor of metalloproteinases 1
MDA	malondialdehyde
MMP9	matrix metalloproteinase 9
MPO	myeloperoxidase
NO	nitric oxide
SP-D	surfactant protein D
